# Moral judgment competence of midwifery students and its influencing factors: a cross-sectional study

**DOI:** 10.3389/fpubh.2025.1626707

**Published:** 2025-09-24

**Authors:** Juan Hu, Min Fan, Hui Ding, Xi Chen

**Affiliations:** ^1^Hunan College of Foreign Studies, Changsha, Hunan, China; ^2^Xiang Zhong Normal College for Preschool Education, Shaoyang, Hunan, China; ^3^The Second Xiangya Hospital of Central South University, Changsha, Hunan, China; ^4^Joint Research Centre for Primary Health Care and School of Nursing, The Hong Kong Polytechnic University, Kowloon, Hong Kong SAR, China

**Keywords:** midwifery students, moral judgment competence, empathy, ethic, education

## Abstract

**Background:**

Midwives frequently encounter complex ethical dilemmas in clinical practice, underscoring the need for strong moral judgment competence. However, limited research has investigated this competence among midwifery students in China.

**Objectives:**

This study aimed to assess the level of moral judgment competence among Chinese midwifery students, examine its relationship with empathy, and identify influencing factors.

**Methods:**

A cross-sectional study was conducted with 213 midwifery students from three vocational colleges in Hunan Province, China. Data were collected using the Chinese versions of the Moral Judgment Test (MJT) and the Jefferson Scale of Empathy for Nursing Students (JSE-NS), along with demographic information. Statistical analyses included Pearson’s correlation and multiple linear regression.

**Results:**

The mean MJT score was 11.81 ± 7.48, indicating a moderate level of moral judgment competence. Empathy also reached a moderate level (87.17 ± 12.90), and a significant positive correlation was found between empathy and moral judgment competence (*r* = 0.502, *p* < 0.01). Multiple linear regression identified five significant predictors: year of study, only-child status, religious affiliation, prior experience in studying nursing ethics, and empathy level, collectively explaining 37.5% of the variance in moral judgment competence (*F* = 22.219, *p* < 0.05, adjusted *R*^2^ = 0.375).

**Conclusion:**

This study provides the first empirical evidence on moral judgment competence among Chinese midwifery students. The findings highlight the importance of integrating empathy cultivation and ethics education into midwifery training. Targeted, individualized teaching strategies may be effective in enhancing students’ ethical reasoning and preparing them for future clinical challenges.

## 1 Introduction

As primary healthcare providers for women during childbirth, midwives play a vital role in promoting reproductive health and ensuring the safety of both mothers and newborns (1–3). However, midwives often encounter complex ethical dilemmas in clinical practice, which can result in significant moral distress (4, 5). Common ethical issues include obtaining informed consent from pregnant women, protecting the privacy of women with infectious diseases, addressing pregnancy termination for non-lethal fetal anomalies, and deciding whether to withhold resuscitation for newborns with severe birth defects (6–9). These situations demand that midwives possess strong moral judgment competence to make sound ethical decisions. A lack of such competence may compromise the quality of care, reduce patient satisfaction, and lead to adverse health outcomes (8, 10–12).

Midwifery students will be future midwives. It is therefore essential that midwifery students develop robust moral judgment competence in preparation for their future roles. This competence enables them to navigate ethical dilemmas in both routine and complex pregnancy care. Moral judgment competence refers to an individual’s ability to evaluate behaviors, events, or situations based on moral principles, discern right from wrong, and make ethically sound decisions (13, 14). It encompasses moral reasoning, identification, and critique, and is recognized as a core competency for healthcare professionals. According to Rest’s Four Component Model—moral sensitivity, moral judgment, moral motivation, and moral character—moral judgment serves as a bridge between moral cognition and ethical behavior (15). This competence is shaped by rational analysis, emotional experience, and sociocultural influences (16), and is significantly affected by the accessibility and quality of ethics education (14, 17).

Understanding the current status and influencing factors of moral judgment competence among midwifery students is critical to designing effective educational interventions. While instruments have been developed to assess this competence, most research has focused on nurses and practicing midwives, with limited quantitative studies targeting midwifery students globally.

In response to population aging, China implemented the “three-child” policy in May 2021, encouraging families to have up to three children (18). This has led to an increase in pregnancies among advanced maternal age (AMA) women and a corresponding rise in high-risk pregnancies, placing greater demands on midwives’ professional capabilities (19). Consequently, midwifery development in China has received growing attention (20). The Ministry of Education’s 2024 Catalogue of Undergraduate Specialties reclassified midwifery as a state-controlled major. Despite this advancement, only 25 universities in China currently offer undergraduate midwifery programs, and the majority of midwifery education remains within vocational colleges (19). While ethics-related content is included in most curricula (21), moral judgment competence is not routinely used as a metric for assessing educational effectiveness. Moreover, little is known about the current level and determinants of moral judgment competence among Chinese midwifery students.

Empathy—the ability to understand, share, and respond to the emotions and needs of others—is a multifaceted construct encompassing emotional, cognitive, moral, and behavioral dimensions (22, 23). Studies have shown that empathetic midwives contribute to more positive childbirth experiences, including reduced perineal trauma and increased maternal satisfaction (23, 24). Furthermore, empathy has been found to directly influence moral sensitivity (25), one of the components of Rest’s Four Component Model (15). Research also suggests that empathy enhances moral judgment, though the mechanisms underlying this relationship remain underexplored (26). Despite the theoretical linkage between empathy and moral judgment, empirical studies examining their association—particularly in midwifery students—are lacking.

Given that empathy is a complex psychological construct shaped by both emotional processes (27) and professional values (28), this study aims to investigate the relationship between empathy and moral judgment competence among midwifery students. Understanding this relationship may inform the development of targeted educational strategies to enhance moral judgment competence in future midwives.

## 2 Methods

### 2.1 Aims

The purposes of this study were to.

1. assess the level of moral judgment competence among Chinese midwifery students;
2. examine the relationship between moral judgment competence and empathy;
3. identify the factors influencing moral judgment competence.

### 2.2 Study design

We conducted a descriptive, cross-sectional study.

### 2.3 Participants and settings

Participants were midwifery students from three higher vocational colleges in Hunan Province, China. A cluster sampling method was employed.

According to Kendall’s sample size estimation method, the required sample size for multiple linear regression should be 10–20 times the number of independent variables (29). In this study, 14 variables were included—moral judgment competence, empathy, and 12 sociodemographic factors. Considering a 20% non-response rate, the estimated final sample size ranged from 175 (14 × 10 ÷ 0.8) to 350 (14 × 20 ÷ 0.8) participants.

Inclusion criteria were as follows:

1. full-time midwifery students enrolled in higher vocational colleges;
2. aged 18 years or older;
3. willing to voluntarily participate in the study.

Exclusion criteria included:

1. students currently on leave of absence or internship;
2. those with a diagnosed mental illness or cognitive impairment that could affect their ability to complete the questionnaire accurately.

### 2.4 Measurements

#### 2.4.1 General information questionnaire

A total of 12 sociodemographic variables were included in this section: gender, age, year of study, place of residence, ethnicity, only-child status, class cadre experience, presence of medical professionals in the family, religious affiliation, prior experience in studying nursing ethics, perceived importance of nursing ethics, and level of fondness for the midwifery major.

#### 2.4.2 Moral judgment test (MJT)

Moral judgment competence among midwifery students was assessed using the Moral Judgment Test (MJT), developed by Lind (30). The MJT has been translated into multiple languages and is widely used in international research. It comprises two moral dilemmas—the worker’s dilemma and the doctor’s dilemma. Dilemma 1 involves workers who illegally enter the administrative offices of a company to obtain evidence supporting an allegation. Dilemma 2 concerns a physician who assists a terminally ill patient in ending her life (euthanasia) at her request. Each dilemma includes 13 items. The first item asks participants to indicate their agreement with the protagonist’s action (e.g., “Do you think the two workers did the right thing?” or “Do you think the doctor did the right thing?”). The remaining 12 items consist of six arguments in favor of and six against the decision. Participants rate each argument on a 9-point scale ranging from −4 (strongly disagree) to +4 (strongly agree), with 0 indicating uncertainty. Responses are used to calculate a consistency score, or C-index, based on the 24 arguments. The C-index ranges from 1 to 100 and reflects the level of moral judgment competence, categorized as low (1–9), medium (10–29), high (30–49), and very high (≥50). The MJT was revalidated for use in the Chinese cultural context by Quan (31), demonstrating high internal consistency, with a Cronbach’s alpha of 0.93.

#### 2.4.3 Jefferson Scale of Empathy for Nursing Students (JSE-NS)

The Jefferson Scale of Empathy-Medical Students (JSE-S) was originally developed by Hojat et al. (32) to assess empathy among medical students. In 2011, Qiu et al. (33) translated it into the Chinese version of the Jefferson Scale of Empathy for Nursing Students (JSE-NS), specifically adapted to measure empathy levels in nursing students. The Chinese version retains the structure of the original scale, comprising 20 items across three dimensions: perspective-taking (10 items), compassionate care (7 items), and standing in the patient’s shoes (3 items).

The JSE-NS is a self-administered instrument using a seven-point Likert-type scale ranging from 1 (“strongly disagree”) to 7 (“strongly agree”). Among the 20 items, 10 are reverse-scored to control for response bias. Total scores range from 20 to 140, with higher scores indicating greater levels of empathy. The Chinese version demonstrated strong internal consistency, with a Cronbach’s alpha of 0.74. According to a classification system used by Jin et al. (23), empathy scores are categorized as low (<56), moderate (56–112), and high (>112).

### 2.5 Date collection

Data collection was conducted between February and April 2024. Participants were recruited from three higher vocational colleges in Hunan Province, China, comprising a total of 407 midwifery students. After explaining the study’s purpose, potential risks, and benefits to both college counselors and students, data were collected using two methods: paper-based questionnaires at one college and electronic questionnaires at the other two colleges via the Wenjuanxing (a tool similar to Typeform). All participants were assured of confidentiality and informed of their right to withdraw at any time.

### 2.6 Data analysis

Data were analyzed using SPSS version 25.0. Descriptive statistics were used to summarize participants’ sociodemographic characteristics, Moral Judgment Test (MJT) scores, and Jefferson Scale of Empathy for Nursing Students (JSE-NS) scores. Pearson’s correlation analysis was conducted to examine the relationship between moral judgment competence and empathy. Independent-samples t-tests and one-way analysis of variance (ANOVA) were used to compare MJT scores across demographic subgroups. Subsequently, multiple linear regression analysis was conducted to identify significant predictors of moral judgment competence, with MJT scores as the dependent variable. Independent variables included the JSE-NS score and sociodemographic variables that met the screening threshold in the univariate analysis. Statistical significance was set at *p* < 0.05.

### 2.7 Validity, reliability, and rigor

To ensure the validity of the questionnaire data, the electronic questionnaire was configured to permit only one submission per IP address. All returned questionnaires were carefully reviewed. Questionnaires exhibiting uniform responses across all items, incomplete entries, or items with more than two selected answers were deemed invalid and excluded. Two research assistants were responsible for downloading and verifying the data from Wenjuanxing and cross-checking the hard copy questionnaires.

## 3 Results

### 3.1 General information characteristics

A total of 225 midwifery students participated in the survey, of which 213 valid questionnaires were obtained, yielding a valid response rate of 94.7%. The mean age of participants was 19.7 years. The majority were female (94.8%). The sample comprised 79 first-year students (37.1%), 63 s-year students (29.6%), and 71 third-year students (33.3%). Table 1 presents the moral judgment competence scores of the midwifery students alongside their complete demographic characteristics.

**Table 1:** Demographic characteristics of participants (*N* = 213).

Characteristics		*N* (%)	Mean ± SD	*t*/*F*	*p*
Gender	Male	11 (5.2)	15.65 ± 12.02	2.076	0.039*
	Female	202 (94.8)	11.51 ± 7.73		
Age (years)	≤19	99 (46.5)	10.83 ± 6.87	2.477	0.086
	20–21	107 (50.2)	12.75 ± 7.93		
	≥22	7 (3.3)	10.31 ± 7.47		
Year of study	1st year	79 (37.1)	10.06 ± 6.58	3.550	0.030*
	2nd year	63 (29.6)	12.85 ± 8.34		
	3rd year	71 (33.3)	12.87 ± 7.35		
Place of residence	Village	133 (62.4)	11.96 ± 7.61	0.064	0.938
	Rural–urban continuum	31 (14.6)	11.54 ± 6.91		
	City	49 (23.0)	11.60 ± 7.62		
Ethnicity	Han	170 (79.8)	11.85 ± 7.76	0.159	0.874
	Ethnic minority	43 (20.2)	11.65 ± 6.33		
Only-child status	Yes	35 (16.4)	9.35 ± 7.02	−2.151	0.033*
	No	178 (83.6)	12.30 ± 7.49		
Class cadre experience	Yes	69 (32.4)	11.67 ± 6.45	−1.024	0.935
	No	144 (67.6)	12.84 ± 8.08		
Presence of medical professionals in the family	Yes	37 (17.4)	11.54 ± 6.58	−0.245	0.806
	No	176 (82.6)	11.87 ± 7.67		
Religious affiliation	Yes	21 (9.9)	14.53 ± 9.17	4.200	0.000*
	No	192 (90.1)	11.07 ± 7.56		
Prior experience in studying nursing ethics	Yes	151 (70.9)	12.57 ± 7.17	2.339	0.020*
	No	62 (29.1)	9.96 ± 7.93		
Perceived importance of nursing ethics	Important	189 (88.7)	11.72 ± 5.43	1.842	0.161
	A neutral attitude	21 (9.9)	11.74 ± 6.84		
	Unimportant	3 (1.4)	11.87 ± 1.41		
Level of fondness for the midwifery major	Like	149 (70.0)	11.71 ± 7.29	0.392	0.676
	A neutral attitude	55 (25.8)	11.75 ± 7.77		
	Unlike	9 (4.2)	12.15 ± 9.20		

SD, standard deviation. *p < 0.05.

### 3.2 Moral judgment competence

**Figure 1:**
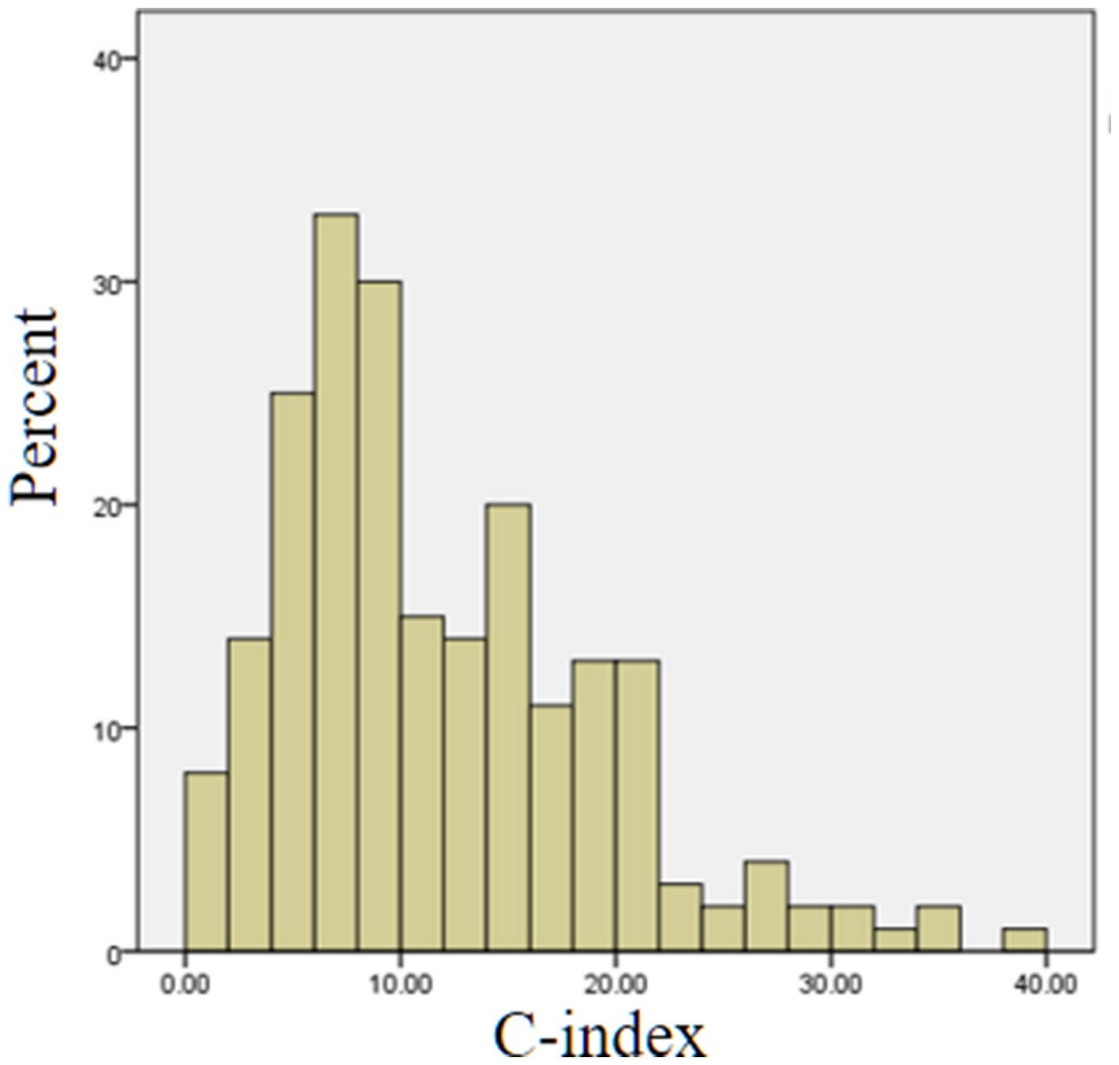
The C-index distribution in Chinese midwifery students (*N* = 213).

The findings showed that the mean C-index score among Chinese midwifery students was 11.81 ± 7.48, indicating a medium level of moral judgment competence (30). However, 51.7% of students demonstrated a low C-index, 45.5% fell within the medium range, and only 2.8% achieved a high level. Notably, none of the students attained a very high C-index (see Figure 1). As shown in Table 2, in response to the worker’s dilemma (stealing evidence), 55.9% of students opposed the worker’s actions, 25.4% were neutral, and 18.7% expressed support. In the doctor’s dilemma (euthanasia), 40.4% of students opposed the doctor’s behavior, 25.8% were neutral, and 33.8% expressed support.

**Table 2:** Analysis of midwifery students’ attitudes regarding the ‘worker’s dilemma’ and ‘doctor’s dilemma (*N* = 213).

Statistical items		−4	−3	−2	−1	0	+1	+2	+3	+4
Worker’s dilemma	Numbers	73	22	16	8	54	21	10	3	6
	Percentage (%)	34.3	10.3	7.5	3.8	25.4	9.8	4.7	1.4	2.8
Doctor’s dilemma	Numbers	56	10	8	12	55	22	19	16	15
	Percentage (%)	26.3	4.7	3.8	5.6	25.8	10.3	8.9	7.5	7.1

Range of scale from −4 to +4 (−4 = strongly disagree, 0 = uncertain, +4 = strongly agree).

### 3.3 Descriptive statistics of JSE-NS

The mean total score on the Jefferson Scale of Empathy for Nursing Students (JSE-NS) among Chinese midwifery students was 87.17 ± 12.90, indicating a moderate level of empathy (23) (see Table 3). Among the three subscales, “Standing in the Patient’s Shoes” received the highest mean score, while “Compassionate Care” received the lowest.

**Table 3:** Chinese midwifery students’ responses on JSE-NS (*N* = 213).

JSE-NSsub-dimensions	No. of item	Total subscale ( $\bar{x}$ ± s)	Minimum	Maximum	Each item ( $\bar{x}$ ± s)
Factor 3: Standing in the patient’s shoes	3	15.18 ± 2.92	4.00	21.00	5.06 ± 0.97
Factor 1: Perspective-taking	10	43.88 ± 6.96	22.00	70.00	4.39 ± 0.70
Factor 2: Compassionate care	7	28.11 ± 5.68	15.00	49.00	4.02 ± 0.81
Total	20	87.17 ± 12.90	44.00	140.00	4.36 ± 0.65

### 3.4 Relationship between moral judgment competence and empathy

Pearson’s correlation coefficients are presented in Table 4. A significant positive correlation was found between the total scores of the Moral Judgment Test (MJT) and the Jefferson Scale of Empathy for Nursing Students (JSE-NS) (*r* = 0.502, *p* < 0.01), indicating that higher levels of empathy were associated with greater moral judgment competence. Additionally, all subscales of the JSE-NS were positively correlated with the MJT scores.

**Table 4:** Correlation coefficients between moral judgment competence and empathy among midwifery students (*N* = 213).

	JSE-NS			
	Overall	Perspective-taking	Compassionate care	Standing in the patient’s shoes
MJT				
Overall	0.502^**^	0.479^**^	0.468^**^	0.169^*^

MJT, Moral Judgment Test; JSE-NS, Jefferson Scale of Empathy for Nursing Students. **p < 0.01, *p < 0.05.

### 3.5 Associated factors

A comparison of moral judgment competence (MJT scores) across various demographic characteristics is presented in Table 1. The analysis identified five factors with statistically significant differences (*p* < 0.05): gender, year of study, only-child status, religious affiliation, and prior experience in studying nursing ethics.

### 3.6 Multiple linear regression analysis results

Table 5 presents the results of the multiple linear regression analysis, with moral judgment competence as the dependent variable. The independent variables included gender, year of study, only-child status, religious affiliation, prior experience in studying nursing ethics, and the total JSE-NS score. The analysis identified five significant predictors of moral judgment competence among Chinese midwifery students: Year of study (*β* = 0.080, *p* = 0.006): Using first-year students as the reference group, each additional academic year was associated with an average increase of 0.080 standard deviations (SD) in moral judgment competence scores. Only-child status (*β* = 0.205, *p* < 0.001): Compared to only-children (reference group), non-only-children had moral judgment competence scores that were, on average, 0.205 SD higher. Religious affiliation (*β* = −0.311, *p* < 0.001): Taking students with religious affiliation as the reference group, students without religious affiliation scored 0.311 SD lower on average. Prior experience in studying nursing ethics (*β* = −0.171, *p* = 0.005): Students without prior experience in studying nursing ethics scored, on average, 0.171 SD lower than those with such experience (reference group). Total JSE-NS score (*β* = 0.405, *p* < 0.001): A one SD increase in the total JSE-NS score was associated with an average increase of 0.405 SD in moral judgment competence. Collectively, these variables explained 37.5% of the variance in moral judgment competence (*F* = 22.219, *p* < 0.05; adjusted *R*^2^ = 0.375).

**Table 5:** Independent predictors for the level of moral judgment competence in Chinese midwifery students (*N* = 213).

Predictors	Description of independent variable assignment	Standardized beta (*β*)	*t*	Sig.
Gender	1 = Male 2 = Female	−0.073	−1.242	0.216
Year of study	1 = 1st year 2 = 2nd year 3 = 3rd year	0.080	1.377	0.006
Only-child status	1 = Yes 2 = No	0.205	3.630	0.000
Religious affiliation	1 = Yes 2 = No	−0.311	−5.220	0.000
Prior experience in studying nursing ethics	1 = Yes 2 = No	−0.171	−2.867	0.005
Total scale score of JSE-NS		0.405	7.122	0.000

*F* = 22.219, *p* < 0.05, Adjusted *R*^2^ = 0.375. The category coded as 1 was used as the reference group.

## 4 Discussion

To the best of our knowledge, this study is the first to investigate the moral judgment competence of midwifery students in China, and the findings may serve as a foundational reference for future research in this area.

In our sample, the mean MJT score among Chinese midwifery students was 11.81 ± 7.48, comparable to those reported for Chinese physical education students (13.41 ± 10.13) (34) and nursing students in the Czech Republic (14.24 ± 9.56) (14). All fall within the 10–29 range, indicating a moderate level of moral judgment competence (30). However, further analysis revealed that only 2.8% of students reached a high level of moral judgment competence, while 51.7% scored low and 45.5% scored at a medium level. These results suggest a clear need to enhance midwifery students’ capacity for moral reasoning.

Regarding students’ responses to the MJT dilemmas, the highest level of opposition (55.9%) was observed in the worker’s dilemma (stealing evidence), with only 18.7% expressing support. This finding may reflect the influence of traditional Chinese cultural values, which emphasize social harmony and the rejection of retaliatory actions such as “an eye for an eye” (34). Such behavior transforms the individual from a rights victim to a rights violator, which is considered morally unacceptable.

In contrast, students’ responses to the doctor’s dilemma (euthanasia) were more divided. Only 40.4% opposed the doctor’s action, while 33.8% supported it. This ambivalence may stem from students’ limited understanding of the ethical and legal implications of euthanasia. Although some may perceive euthanasia as a compassionate act to relieve suffering, it remains legally prohibited in China. These findings suggest that while students exhibit strong opposition to overtly illegal acts like theft, their ethical reasoning becomes less clear in the face of more morally complex or controversial issues. This underscores the urgent need to strengthen ethics education in midwifery programs.

To address this gap, ethics education should be improved in the following ways: (1) strengthening the teaching of professional codes and relevant regulations; (2) fostering foundational ethical concepts and reasoning skills to ensure sound moral judgment; and (3) encouraging deeper reflection on complex ethical issues through ethics-focused discussions, case analysis, and experiential learning to enhance moral sensitivity and critical thinking.

Our findings also revealed a significant positive correlation between empathy and moral judgment competence (*r* = 0.502, *p* < 0.01), with multiple linear regression identifying empathy as a key predictor. This suggests that enhancing empathy may be an effective way to improve moral judgment competence. Prior research has recognized empathy as a crucial component of effective healthcare practice (35, 36).

Despite this, the mean JSE-NS score among midwifery students was 87.17 ± 12.90, indicating a moderate level of empathy, similar to scores found in Chinese vocational nursing students (37). Among the three JSE-NS subscales, “Compassionate Care” received the lowest score, suggesting difficulty in responding empathetically to others’ emotions. This may be attributed to the students’ limited clinical exposure and social experience. Empathy is shaped by both innate personality traits and acquired skills (38, 39), implying that it can be effectively cultivated through targeted educational interventions.

Midwifery educators should consider incorporating humanistic courses (e.g., “Interpersonal Communication”), embedding empathy development into the curriculum, and using diverse pedagogical strategies such as scenario-based simulations, role-playing, and standardized patients. Additionally, increasing opportunities for students to engage meaningfully with patients during clinical placements and involving them in psychological care and emotional support may further foster empathy and, in turn, strengthen moral judgment competence.

Beyond empathy, significant differences in moral judgment competence were observed based on several demographic characteristics. First, competence increased significantly with academic progression, consistent with the findings of Auvinen (40). Senior students demonstrated higher levels of moral judgment competence, reflecting the positive impact of academic advancement and clinical experience. However, this progression is not automatic. Therefore, early integration of clinical practice and simulation-based teaching could help bridge theoretical knowledge with practical application, thereby enhancing students’ ethical awareness and judgment.

Second, students from non-only-child families scored higher in moral judgment competence compared to their only-child counterparts. Students with siblings may develop greater interpersonal awareness, empathy, and respect for others’ perspectives due to shared familial attention and early socialization. In contrast, only-child students, often raised in more protective environments, may exhibit higher levels of self-centeredness (41). Educators should pay particular attention to cultivating empathy and patient-centered thinking in only-child students to promote moral development.

Third, religious affiliation emerged as a significant predictor of moral judgment competence, aligning with the findings of Hone (42). Students with religious beliefs scored higher, possibly because many religious values emphasize compassion, care, and ethical responsibility (43). Individuals with stronger religious commitments are also less likely to adopt utilitarian approaches in moral reasoning (44). In China, traditional moral values are influenced by diverse religious traditions such as Buddhism, Taoism, and Confucianism (45), all of which contribute to moral development in different ways.

Finally, prior experience in studying nursing ethics was positively associated with higher moral judgment competence, supporting the findings of Kim et al. (46). Students who had completed ethics coursework demonstrated stronger ethical reasoning skills. These findings reinforce the value of ethics education as a core component of professional training for midwifery students. In line with the requirements established by national regulatory bodies such as the Ministry of Education and the National Health Commission, most Chinese institutions now include ethics courses in midwifery programs to equip students with the ability to identify ethical issues, resolve moral dilemmas, and uphold patient-centered care.

However, variations exist in the depth, breadth, and instructional methodologies of ethics training between vocational and undergraduate programs, reflecting their distinct professional objectives. Vocational education primarily prepares midwives for roles in primary healthcare settings (e.g., community health centers and county-level hospitals), whereas undergraduate education focuses on training midwives for tertiary hospitals and preparing graduates for leadership and management roles in nursing. Consequently, notable differences in the approach to ethics education have emerged across these pathways. Undergraduate programs generally provide a more comprehensive and theoretical foundation, emphasizing critical thinking and advanced ethical reasoning. In contrast, vocational programs—while equally committed to ethical practice—tend to focus on the practical application of established ethical principles to real-world clinical scenarios encountered in midwifery. These distinctions in educational focus, arising from the differing levels of training, may influence how students develop and apply moral judgment competence in clinical practice. Nevertheless, the central conclusion remains clear: institutionalized ethics education initiatives, despite variations across educational pathways, significantly enhance students’ moral judgment competence. This underscores the fundamental importance of ethics education as a core component of professional midwifery training in China.

### 4.1 Limitations

This study has three main limitations. First, a major limitation of this study is the issue of sample representativeness and gender imbalance. The sample was drawn from three higher vocational colleges within a single province (Hunan), which restricts the generalizability of the findings. Moreover, over 94.8% of participants were female, precluding meaningful gender-based comparisons and limiting the interpretation of potential gender effects. Future research should aim to recruit a more diverse and representative sample, including both vocational and undergraduate midwifery students from multiple regions across China. Efforts should also be made to include an adequate number of male students to allow for statistically valid gender-based analyses. Second, this study is limited by its exclusive reliance on quantitative methods within a constrained time frame, which may not adequately capture the complex and multifaceted nature of moral judgment competence. Additionally, the use of dual administration modes (paper-based and electronic) for the MJT and JSE-NS introduces potential measurement effects. Although standardized instructions were provided and data collection was conducted simultaneously, variations in response patterns related to mode-specific factors—such as attentional focus, response bias, or environmental context—may still have occurred. Future research should employ a single mode of administration to eliminate this confounding variable and consider adopting mixed-methods approaches to gain deeper and more comprehensive insights. Third, given that moral development is a dynamic, time-dependent process, the cross-sectional design of this study—capturing data at a single time point—cannot establish the causal direction between moral judgment competence and empathy. Future research should adopt longitudinal designs to systematically track the development of moral judgment competence among midwifery students, thereby helping to clarify the causal mechanisms through which empathy may influence ethical development.

## 5 Conclusion

This study is the first to explore the moral judgment competence of midwifery students in China and provides important insights into its current status and influencing factors. The findings reveal that while most students exhibit low to moderate levels of moral judgment competence, there is considerable room for improvement—particularly in addressing ethically complex issues such as euthanasia. The significant positive association between empathy and moral judgment competence underscores the potential of empathy-focused education to enhance students’ ethical reasoning abilities. In addition, factors such as year of study, only-child status, religious affiliation, prior experience in studying nursing ethics were found to influence moral judgment competence, suggesting the need for targeted pedagogical interventions. Based on these findings, vocational curriculum designers could consider establishing a dedicated “Nursing Humanities and Social Sciences Module,” incorporating core courses such as nursing ethics, healthcare laws and regulations, interpersonal communication, nursing humanities, and nursing psychology.

Furthermore, midwifery education should prioritize the integration of ethics and empathy training through diversified teaching strategies, including scenario-based simulations, reflective practices, and clinical exposure. Given that midwifery students in China are eligible to take the National Nurse Licensure Examination, national nursing authorities should consider placing greater emphasis on humanities-related content within the examination framework. This adjustment would help reinforce the importance of ethical and humanistic competencies at the entry level of professional nursing practice. Strengthening students’ moral and humanistic development is essential for preparing them to navigate ethical challenges in maternal and neonatal care and for fostering professional, compassionate, and ethically responsible midwives.

## Author’s note

We failed to secure permission from the copyright holder before using the JSE in our project. After contacting the copyright holder, scoring methods and use of the scale were verified and used without modification. Retrospective permission has been received from Thomas Jefferson University.

## Data Availability

The datasets used and/or analyzed during the current study are available from the corresponding author on reasonable request.
